# Psychobiotics and fecal microbial transplantation for autism and attention-deficit/hyperactivity disorder: microbiome modulation and therapeutic mechanisms

**DOI:** 10.3389/fcimb.2023.1238005

**Published:** 2023-07-24

**Authors:** Min-jin Kwak, Seung Hyun Kim, Hoo Hugo Kim, Rahul Tanpure, Johanna Inhyang Kim, Byong-Hun Jeon, Hyun-Kyung Park

**Affiliations:** ^1^ Department of Agricultural Biotechnology and Research Institute of Agriculture and Life Science, Seoul National University, Seoul, Republic of Korea; ^2^ Department of Pediatrics, Hanyang University College of Medicine, Seoul, Republic of Korea; ^3^ Department of Earth Resources and Environmental Engineering, Hanyang University, Seoul, Republic of Korea; ^4^ Department of Psychiatry, Hanyang University Medical Center, Seoul, Republic of Korea; ^5^ Clinical Research Institute of Developmental Medicine, Hanyang University Hospital, Seoul, Republic of Korea

**Keywords:** autism spectrum disorder, attention-deficit/hyperactivity disorder, psychobiotics, fecal microbial transplantation, gut microbiome

## Abstract

Dysbiosis of the gut microbiome is thought to be the developmental origins of the host’s health and disease through the microbiota-gut-brain (MGB) axis: such as immune-mediated, metabolic, neurodegenerative, and neurodevelopmental diseases. Autism spectrum disorder (ASD) and attention-deficit/hyperactivity disorder (ADHD) are common neurodevelopmental disorders, and growing evidence indicates the contribution of the gut microbiome changes and imbalances to these conditions, pointing to the importance of considering the MGB axis in their treatment. This review summarizes the general knowledge of gut microbial colonization and development in early life and its role in the pathogenesis of ASD/ADHD, highlighting a promising therapeutic approach for ASD/ADHD through modulation of the gut microbiome using psychobiotics (probiotics that positively affect neurological function and can be applied for the treatment of psychiatric diseases) and fecal microbial transplantation (FMT).

## 1 Introduction

Two childhood-onset neurodevelopmental disorders that have been linked to gut microbial dysbiosis are autism spectrum disorder (ASD) and attention-deficit/hyperactivity disorder (ADHD) (Prosperi et al., 2022). ASD and ADHD are highly prevalent, commonly co-occur with each other, and share overlapping symptoms (Bundgaard-Nielsen et al., 2023). Both have been found to be related to environmental exposures in early life, like endocrine disrupting chemicals (EDC), and EDCs have been suggested to induce microbiota changes through the gut-brain-microbiota axis conferring susceptibility to neurodevelopmental disorders (Ramírez et al., 2022). In the early twenty-first century, many papers highlighted the connection between the brain and gut. The term “psychobiotics”, defined as the probiotic bacteria-derived molecules exerting psychological potential to support mental health by targeting microbial interventions, was newly coined in 2013. The therapeutic potential of psychobiotics ranges from mood changes and anxiety to neurodegenerative diseases and neurodevelopmental disorders (Sharma et al., 2021).

ASD is characterized by impairments in behavioral domains such as social communication, restricted interests, and repetitive behavior. Although the high heritability of ASD suggests that genetics is a key factor in its pathogenesis (Tick et al., 2016). Gene-environment interactions have also been reported to be substantially involved, with estimates that more than 50% of neurobiology is driven by non-heritable factors (Cheroni et al., 2020). ADHD is defined as a neurodevelopmental disorder, and it is described by hyperactivity, inattention, and excessive impulsiveness. The pathogenesis of ADHD is complex, and representative factors have been investigated including genetic, environmental, and perinatal damage-associated factors (Kalenik et al., 2021). ASD and ADHD are highly comorbid, where 20-50% children with ADHD meet the criteria for ASD and 30-80% of ASD meet the criteria for ADHD (Rommelse et al., 2010). High co-occurrence rate challenges differential diagnosis and also worsens symptom severity and prognosis (Zhou et al., 2023). Based on this high comorbidity, extensive research has been conducted on the overlapping genetic factors and shared biological underpinnings of ASD and ADHD (Liu et al., 2020).

**Figure 1:**
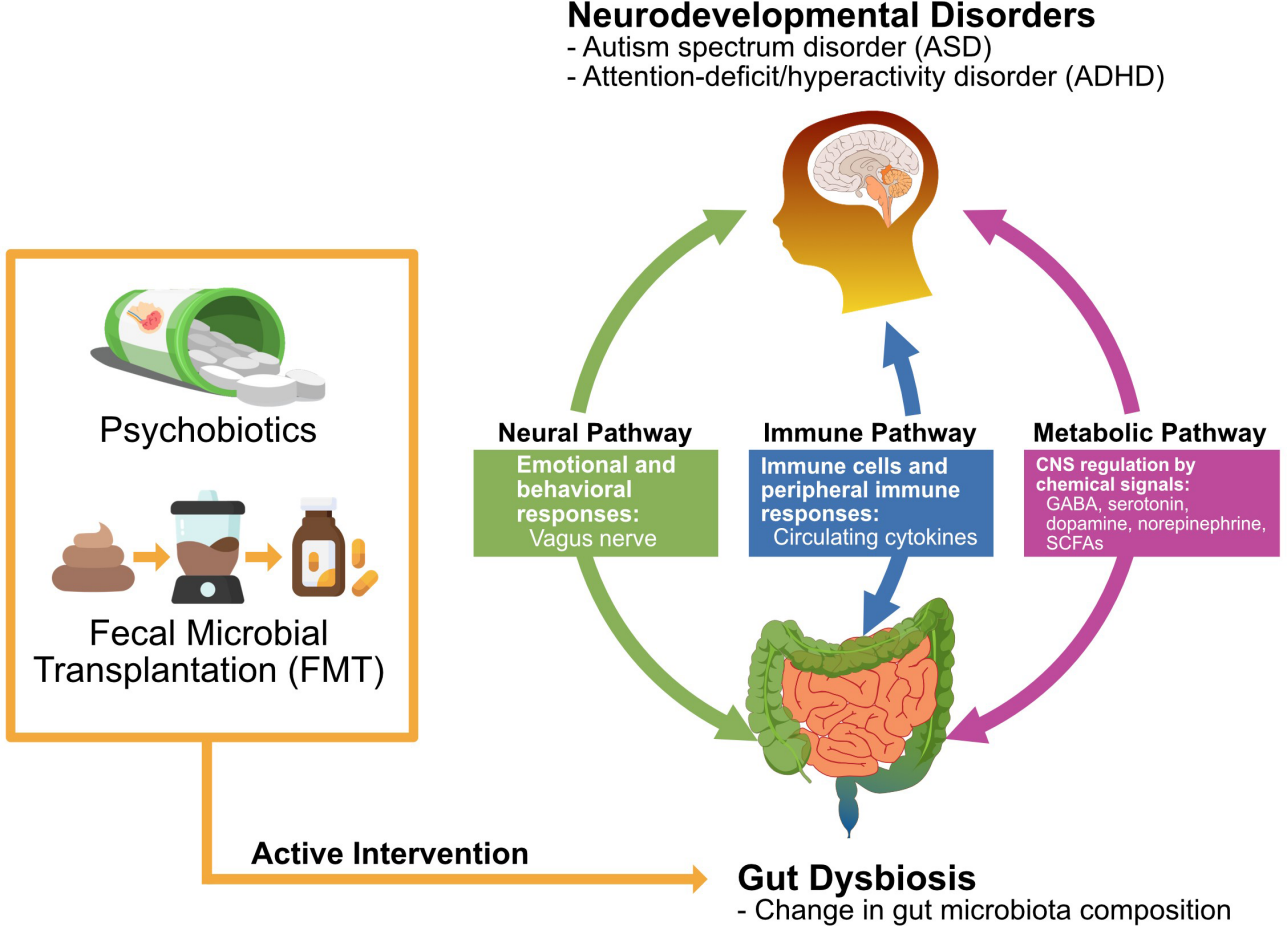
Action mechanisms by which psychobiotics and fecal microbial transplantation exert the potential therapeutic effect on ASD and ADHD. Abbreviations: γ-aminobutyric acid (GABA), short-chain fatty acids (SCFAs).

Here, we reviewed the literature on the influence of psychobiotics and fecal microbial transplantation (FMT) on the gut microbiome and behaviors/gastrointestinal (GI) symptoms related to ASD and ADHD, and the relationship between neurodevelopmental disorders and psychobiotics has received considerable attention in recent years. There is a bidirectional interaction in the microbiota-gut-brain (MGB) axis, and its modulation exerts beneficial effects on brain activity and behavior as potential treatments (Bundgaard-Nielsen et al., 2020). In light of these considerations, the gut-brain axis is an attractive target for developing novel therapeutics, such as the use of probiotics, for neurodevelopmental disorders (Sarkar et al., 2016). The main subject of our review is the commonly known gut microbiome in ASD and ADHD individuals, and its impact on the symptoms of these neurodevelopmental disorders. And we will also discuss the theoretical basis of the correlations and the therapeutic possibility of psychobiotics and FMT on neurodevelopmental disorders (**Figure 1**).

To the best of our knowledge, no systematic review of randomized controlled trials (RCTs) has been conducted so far, and there are relatively few clinical studies identifying the therapeutic effects of psychobiotics and FMT on ASD and ADHD. Therefore, we thoroughly summarized the latest reports on potential therapeutic mechanisms and promising perspectives, in addition to observed changes in the gut microbiome composition and metabolites. Moreover, this review presents directions for future treatments that could be employed to directly manipulate gut microbiota during early life stages in humans to prevent the development of such diseases.

## 2 Role of gut microbiome axis

### 2.1 Early-life gut microbial colonization and development

**Figure 2:**
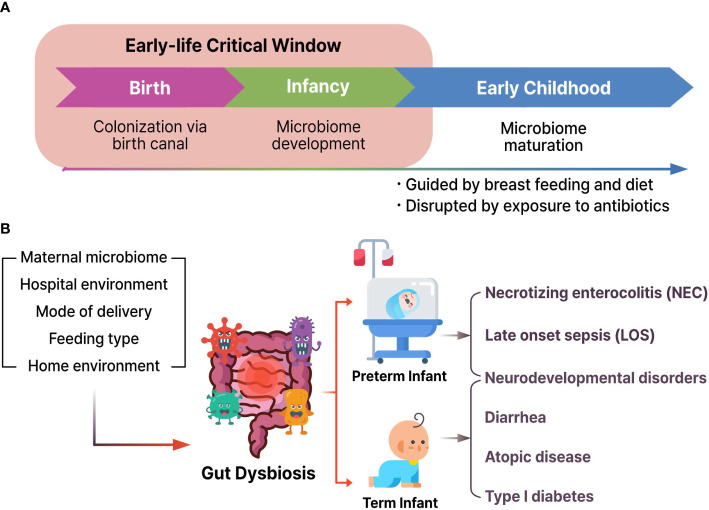
**(A)** Microbial colonization and development from birth to child. **(B)** Various environmental factors could induce gut dysbiosis and result in the pathogenesis of necrotizing enterocolitis, late onset sepsis, neurodevelopmental disorders, diarrhea, atopic disease, and type I diabetes.

Human microbial colonization begins in the fetus and continues to develop and modulate species abundance for approximately three years until the gut microbiome becomes adult-like (**Figure 2A**) (Arrieta et al., 2014; Senn et al., 2020). There is increasing evidence that the gut microbiota and its byproducts could play pivotal functions in the immune system maturation, development, and behavior of the host throughout the life cycle (Erkosar et al., 2013).

Microbiota development follows typical timely changes and the interplay between the gut microbiome and the rest of the human body that have been analyzed through metagenomics studies and recent strain-level profiling (Bäckhed et al., 2015; Selma-Royo et al., 2020). Especially microbial colonization of the newborn period is a critical process that affects long-term neurological outcomes and later-life health (Stiemsma and Michels, 2018; Niemarkt et al., 2019; Korpela et al., 2020).

### 2.2 Gut-brain axis and bidirectional communication

The past 15 years have seen the emergence of the microbiota as one of the critical regulators of gut-brain function through a complex network of signaling pathways, which has led to the appreciation of the importance of a distinct MGB axis (Cryan et al., 2019). There are various bidirectional communicating pathways between the gut microbiome and the brain, which include vagus nerve (VN), immunity with tryptophan metabolism, endocrine system, and enteric nervous system (ENS) with diverse bacterial byproducts, such as peptidoglycans, short-chain fatty acids (SCFAs), and branched-chain amino acids (Jena et al., 2020).

Bidirectional communication between the microbiota and the host through the gut-brain axis is an essential pathway for accessing the synergetic mechanism to modulate the host brain and behavior (Dinan and Cryan, 2017; Ronan et al., 2021). Studies to identify and examine the MGB axis have used different yet complementary microbiota interventions, including germ-free rodents, antibiotic-induced depletion, prebiotic/probiotic supplementation, gastrointestinal infection, and FMT (Cryan et al., 2019). Top-down signaling influences the motor, sensory, and secretory functions of the gastrointestinal tract *via* the efferent fibers of the VN. Bottom-up communication affects the function of the brain, especially the amygdala and hypothalamus, *via* the afferent vagal fibers.

## 3 Neurodevelopmental disorders and gut microbiome

### 3.1 Gut dysbiosis in neurodevelopmental disorders

Dysbiosis may play a role in the etiology and development of neurodevelopment disorders (Nagpal and Cryan, 2021). Earlier reports confirmed that the index of gut microbial α-diversity of 1-year-old children showed a close correlation with cognitive functions at 2-year-old (Carlson et al., 2018). Furthermore, additional human studies have suggested that the first year of life is the most authoritative period in human cognitive development (**Figure 2B**). These results indicate that the occurrence risk of diseases might be increased during fetal development and early life stages.

### 3.2 ASD and gut microbiome

In general, ASD patients frequently coincidently have various gastrointestinal disorders, and it might be due to the tight association between gut microbial disturbances and mental health. Moreover, these connections in ASD individuals have suggested increased gut permeability, called “leaky gut”. If gut permeability arose, the gut barrier allows to cross the bacterial metabolites into animal’s body, and it could negatively affect neurodevelopment during early childhood through the gut-brain axis. Fowlie and colleagues demonstrated that psychobiotics treatment in ASD patients has a potential to relieve ASD symptoms by modulation of the gut microbiome (Fowlie et al., 2018).

An important feature of ASD is its marked comorbidity with gastrointestinal symptoms. A high rate of ASD patients, ranging from 9% to 90%, report comorbid gastrointestinal symptoms such as constipation, abdominal pain, diarrhea, gas, and vomiting (Vuong and Hsiao, 2017). Moreover, the observed GI disturbances were strongly correlated with ASD severity. These GI problems suggest that the intestine plays an important role in ASD pathogenesis. Previous studies on microbiota composition in patients with ASD have shown highly heterogeneous results between studies, but the majority of them have found that the overall microbiota composition of ASD cases is different from that of the controls (Bundgaard-Nielsen et al., 2020). However, no specific bacteria are consistently associated with ASD diagnosis or severity in the literature.

Specifically for ASD, the specific composition of microbial taxa in the human gut, including *Firmicutes*/*Bacteroidetes* ratio, is reported to differ between the control and patient groups, with *Fusobacteria* and *Verrucomicrobia* abundances being lower in the patient group (De Angelis et al., 2013). Microbial taxa have specific roles in the production of substances, such as SCFAs, which are reported to have diverse neurobiological correlations in the context of the MGB axis, and 4-ethylphenylsulfate, which is a dietary tyrosine metabolite that is considered to induce ADS-like behavior among many others (Hsiao et al., 2013; Dalile et al., 2019). In an experiment with genetically engineered mice, maternal interleukin-17α secreted by Th17 cells was observed to induce behavioral and cortical issues in their offspring, suggesting a possible role for the cytokine receptor interleukin-17α in the modulation of ASD (Choi et al., 2016).

### 3.3 ADHD and gut microbiome

ADHD is a common childhood-onset neurodevelopmental disorder that persists into adulthood, with a worldwide prevalence of 5% (Polanczyk et al., 2007). ADHD is distinguished by symptom domains of inattention, hyperactivity, and/or impulsivity. Although some children might not reach the threshold of full diagnosis, ADHD traits are continuously distributed throughout the population (Brikell et al., 2021). ADHD is a complex genetic disorder with a high heritability rate of 76%; however, this estimate encompasses gene by environment interaction and such interactions may account for much of the etiology of ADHD (Faraone and Larsson, 2019). Diverse environmental factors, including perinatal factors (prematurity, low birth weight) and psychosocial determinants (adoption, child neglect), have been reported as reasonable factors to ADHD.

However, there are few reports on the role of the gut microbiome in ADHD patients. A recent systematic review found that all six included studies had distinct taxon findings between patients with ADHD and healthy controls. However, results varied between studies, and there was minimal consensus on which bacterial taxa correlated most with ADHD (Sukmajaya et al., 2021).

In the case of ADHD, there have been multiple attempts to find relationships between clinical features and differences of this disorder and healthy samples based on the gut-brain axis concept. A study that attempted to associate gut microbiota and plasma cytokine levels with ADHD showed a higher abundance of three genera (*Agathobacter*, *Anaerostipes*, and *Lachnospira*) and decreased levels of TNF-α in the ADHD group compared with that of the control group (Wang et al., 2022). However, the studies conducted so far have not been able to show clear relationships between microbial taxa and ADHD, compared to the relatively more established ASD studies (Bundgaard-Nielsen et al., 2020). Considering the relative paucity of scientific literature, more research efforts to clarify the possible relationship between ADHD and the gut-brain axis are required.

## 4 Psychobiotics: potential roles on MGB axis as treatment target

Psychobiotics, next-generation probiotics (NGPs) for the brain, are a special class of probiotics that positively affect neurological function and can be applied for the treatment of psychiatric diseases (Cheng et al., 2019). They are different from typical probiotics in their ability to affect the gut-brain axis by modulating microbial composition, immune activation, VN signaling, and production of neuroactive metabolites, such as neurotransmitters, cytokines, SCFAs, and enteroendocrine hormones (Bermúdez-Humarán et al., 2019; Kwak et al., 2021; Morais et al., 2021). Considering this potential, psychobiotics have a wide-ranging application spectrum from stress alleviation to being an adjuvant in the treatment of diverse neuro-developmental and degenerative diseases (ADHD, ASD, Parkinson’s disease, and Alzheimer’s disease). Generally, conventional psychobiotic bacteria belong to the family *Lactobacilli*, and *Bifidobacteria* (Sharma et al., 2021). A summarized overview of clinical studies on the use of psychobiotics and FMT in individuals with ASD or ADHD is shown in **Table 1**.

**Table 1:** Treatment trials with psychobiotics and fecal microbial transportation in ASD and ADHD patients^1^.

Types	Population	Method	Strain name	Dose	Main effects	Reference
ASD	85 patients (3–6 years)	Probiotics	*S. thermophilus, B. breve*, *B. lungum, B. infantis*, *L. acidophilus, L. plantarum*, *L. paracasei, L. delbrueckii*	9.0 × 10^11^ CFU in 1^st^ month 4.5 × 10^11^ CFU in following	ASD symptoms ↓ Inflammation, ↓ Oxidative stress ↓	(Santocchi et al., 2020)
ASD	131 patients (4–11 years)	Probiotics	*L. plantarum* PS128	3.0 × 10^10^ CFU BW < 30 kg 6.0 × 10^10^ CFU BW > 30 kg	Improve intestine ASD symptoms ↓	(Mensi et al., 2021)
ASD	35 patients (3–25 years)	Probiotics	*L. plantarum* PS128	6.0 × 10^10^ CFU	ASD symptoms ↓ *Veillonella*, ↑ *Streptococcus* ↑	(Kong et al., 2021)
ASD	80 patients (7–15 years)	Probiotics	*L. plantarum* PS128	3.0 × 10^10^ CFU	ASD symptoms ↓	(Liu et al., 2019)
ASD	22 patients (4–10 years)	Probiotics	*L. acidophilus*	5.0 × 10^9^ CFU	ASD symptoms ↓ Urinary arabinitol ↓	(Kałużna-Czaplińska and Błaszczyk, 2012)
ASD	30 patients (5–9 years)	Probiotics	*B. longum, L. rhamnosus*, *L. acidophilus*	5.0 × 10^8^ CFU	ASD symptoms ↓ Gut symptoms ↓ *Bifidobacterium* ↑ *Lactobacillus* ↑	(Shaaban et al., 2018)
ASD	1 patient (12 years)	Probiotics	*B. breve, B. longum, B. infantis*, *L. acidophilus, L. plantarum*, *L. paracasei, L. bulgaricus*, *L. delbrueckii, S. thermophilus*, *S. salivarius*	9.0 × 10^10^ CFU in *Bifido.* 8.0 × 10^10^ CFU in *Lacto.* 2.0 × 10^11^ CFU in *Strepto.*	ASD symptoms ↓ Gut symptoms ↓	(Grossi et al., 2016)
ASD	41 patients (4–11 years)	Prebiotics	Galactooligosaccharide	1.8 g for 6 months	Behavior improved *Bifidobacterium* ↑	(Grimaldi et al., 2018)
ASD	8 patients (2–11 years)	Synbiotics	*B. infantis* Bovine colostrum	2.0 × 10^11^ CFU 5.0– 10.0 g/day	Behavior improved Gut symptoms ↓	(Sanctuary et al., 2019)
ASD	13 patients (3–12 years)	Probiotics	*L. casei, L. plantarum*, *L. acidophilus, L. delbrueckii* *B. lungum, B. infantis, B. breve*, *S. thermophilus*	1.8 × 10^6^ – 3.2 × 10^6^ CFU	Behavior improved Gut symptoms ↓	(Arnold et al., 2019)
ASD	61 patients (2–16 years)	Probiotics	*L. fermentum, L. plantarum*, *L. salivarius* DSM 22	1.0 × 10^10^ CFU	Behavior improved Gut symptoms ↓	(Guidetti et al., 2022)
ASD	18 children (7–17 years)	FMT	Standardized human gut microbiota (Hamilton, Weingarden et al., 2012)	2.5 × 10^12^ cells/day	ASD symptoms ↓	(Kang et al., 2017)
ASD	18 children (7–17 years)	FMT	Standardized human gut microbiota (Hamilton, Weingarden et al., 2012)	2.5 × 10^12^ cells/day	ASD symptoms ↓ Improved behavior	(Kang et al., 2019)
ASD	40 children (3–17 years)	FMT	Standardized human gut microbiota (Hamilton, Weingarden et al., 2012)	2.5 × 10^12^ cells/day	ASD symptoms ↓ Improved behavior	(Li et al., 2021)
ADHD	132 infants (2–13 years)	Probiotics	*L. rhamnosus* GG	1.0 × 10^10^ CFU	ADHD symptom ↓	(Pärtty et al., 2015)
ADHD	35 patients (4–17 years)	Probiotics	*L. rhamnosus* GG	1.0 × 10^10^ CFU	Improve QoL Improve cytokines	(Kumperscak et al., 2020)
ADHD	30 patients (4–16 years)	Probiotics	*B. bifidum* Bf-688	5.0 × 10^9^ CFU	ADHD symptom ↓ Weight gain ↑	(Wang et al., 2022)
ADHD	66 patients (5–55 years)	Synbiotics	*L. mesenteroides, L. paracasei*, *L. plantarum* B-glucan, inulin, pectin, starch	4.0 × 10^11^ CFU 2.5 g of prebiotics	ADHD symptom ↓	(Skott et al., 2020)
ADHD	38 patients (6–12 years)	Probiotics	*B. subtilis, B. bifidum, B. breve*, *B. infantis*, *B. longum*, *L. acidophilus, L. delbrueckii*, *L. casei*, *L. plantarum L. lactis*, *L. salivarius, S. thermophiles*	2.0 × 10^9^ CFU	ADHD symptom ↓	(Ghanaatgar et al., 2022)
ADHD	34 patients (8–12 years)	Probiotics	*L. reuteri, L. acidophilus*, *L. fermentum, B. bifidum*	8.0 × 10^9^ CFU	ADHD symptoms ↓ Inflammation ↓ Oxidative stress ↓	(Sepehrmanesh et al., 2021)
ADHD	1 patient (22 years)	FMT	Healthy doner’s microbiota	Not applicable	ADHD symptom ↓ *F. prausnitzii* ↑ *B. longum* ↓	(Hooi et al., 2022)

^1^Abbreviations: autism spectrum disorders (ASD), attention deficit/hyperactivity disorder (ADHD), fecal microbial transplantation (FMT).

### 4.1 Therapeutic mechanisms/effects on ASD

Gut dysbiosis in ASD has been reported in numerous studies (Kho and Lal, 2018). Patients with ASD possess significantly altered gut microbiota, resulting in GI symptoms. When dysbiosis occurs in disorders such as irritable bowel disease and ASD, the psychobiotics would help the gut microbiota return to normal levels and have positive effects on psychiatric diseases. Consequently, various studies demonstrated that the use of psychobiotics for ASD individuals suffering from GI disorders would be a supplementary therapeutic method (Zheng et al., 2020).

#### 4.1.1 Shift of gut dysbiosis toward eubiosis

##### 4.1.1.1 Affecting the gut microbiome population

One study found reduced D-arabinitol levels, a metabolite of *Candida* species, in the urine of children with ASD after probiotic supplementation (Shaw et al., 1995). This result suggests that probiotics may prevent gastrointestinal colonization by *Candida* species. In another study, the levels of *Bifidobacterium* (known as beneficial bacteria, such as *Lactobacillus* species) were significantly lower in the stool of children with ASD (Shaaban et al., 2018). After probiotic supplementation, there was a significant increase in the colony counts of *Bifidobacterium* and *Lactobacillus* with significant improvement in the severity of ASD and gastrointestinal symptoms. A study in 2015 reported the effect of mixed probiotic administration on gut microbiota composition in children with ASD (Tomova et al., 2015). The abundance of *Clostridia* and *Desulfovibrio* and *Bacteroidetes*/*Firmicutes* ratio were related to the severity of ASD and gastrointestinal symptoms. After probiotics treatment, the amount of *Firmicutes* significantly decreased, which resulted in an increase in the *Bacteroidetes*/*Firmicutes* ratio to a level similar to that observed in healthy children, *Bifidobacterium* increased, and *Desulfovibrio* decreased significantly. Moreover, a study of a mixture of *Lactobacillus* spp. and *Bifidobacterium* spp. in two different rodent ASD models indicated that a probiotic mixture could improve social behavioral symptoms by modulating the gut microbial population (Mintál et al., 2022).

#### 4.1.2 Anti-inflammation and immunomodulation

##### 4.1.2.1 Psychobiotics as immunomodulators

Psychobiotics have a potential to not only reconstruct the gut barrier function by resisting harmful bacteria, but also exert an immunomodulatory effect by reducing circulating hormones and pro-inflammatory cytokines in serum. The gut microbiota has been demonstrated to serve as a regulator of intestinal, systemic, and CNS resident immune cell function (Zheng et al., 2020). Gut microbiota can communicate with the CNS by regulating intestinal and peripheral immune cells and peripheral immune responses *via* circulating cytokines (Arrieta and Finlay, 2012).

##### 4.1.2.2 Psychobiotics reduce inflammation

Any peripheral inflammatory event induces VN to cause the suppression of the release of proinflammatory cytokines from intestinal macrophages (Daliri et al., 2016). Probiotics reduce gut inflammation through various mechanisms, such as reducing inflammatory cytokines and other immunomodulatory effects. For example, anti-inflammatory cytokines (IL-4 and IL-10) and proinflammatory cytokines (TNF-a, IL-1b, IL-2, IL-6, IL-8, IL-12, IL-17, and IL-18) are significantly changed by *Lactobacillus rhamnosus GG* (LGG) (Miyazawa et al., 2015; Cicenia et al., 2016; Fong et al., 2016; Wang et al., 2016; Aoki-Yoshida et al., 2017; Clarke, 2018; Cai et al., 2019).

##### 4.1.2.3 Inflammatory cytokines change in ASD

In clinical studies, Tomova et al. found that TNF-α levels were strongly correlated with GI symptoms and showed a trend toward correlation with ASD severity (Tomova et al., 2015). Probiotic supplementation significantly decreased TNF-α levels in the feces of children with ASD. Similar to this study, Sanctuary demonstrated that psychobiotic supplementation could reduce the intracellular expression of certain cytokines in CD4+ T cells (Sanctuary et al., 2019). The frequency of CD4+/IL-13+ T cells was significantly lower after the treatment.

##### 4.1.2.4 Animal studies on the anti-inflammatory effects of psychobiotics

Adıgüzel et al. demonstrated that dietary treatment with multispecies probiotics formulations (*Sptreptococcus thermophilus*, *Bifidobacterium breve*, *B. animalis*, *Lactobacillus helveticus*, *L. plantarum*, *L. acidophilus*, and *L. paracacei*) attenuated the inflammatory responses in a VPA-induced rodent ASD model. In particular, this study also showed that psychobiotic treatment decreased serum pro-inflammatory cytokine, IL-6 levels, and increased anti-inflammatory cytokine, IL-10 levels, with the improved status of diverse behavior tests including social interaction, anxiety, and repetitive behaviors (Adıgüzel et al., 2022). Alonazi also demonstrated that dietary psychobiotic supplementation could reduce levels of various serum inflammatory cytokines, such as IL-1β, IL-8, IL-10, and IFN-γ, in an ASD rat model induced by a neurotoxic dose of propionic acid (Alonazi et al., 2022).

#### 4.1.3 Neural pathway and chemical signaling

##### 4.1.3.1 Changing microbial signals (neuroendocrine signaling)

The gut microbiota influences the brain directly through neural pathways, including the VN and ENS. The VN connects the ENS and CNS and it could be activated by cytokines, which could be modulated by bacteria, and byproducts from bacteria including endotoxins and peptides. In particular, the neuropeptide could be sensed by receptors associated with dendritic cells in the gut, which could transfer the signals to the brain (Perez-Burgos et al., 2013). Psychobiotics modulate CNS-related behaviors through the VN pathway and the physiological response of various metabolites, including SCFAs, enteroendocrine hormones, cytokines, and neurotransmitters (Bravo et al., 2011; Dinan et al., 2014; Sgritta et al., 2019).

##### 4.1.3.2 Hormones and metabolic changes

The levels of oxytocin and DHEA-S, which have been considered to be etiologies of ASD, were significantly lower in the plasma of children with ASD in a clinical study, and there was a trend towards a correlation between decreased DHEA-S levels and a lower *Bacteroidetes*/*Firmicutes* ratio which increased after probiotic implementation (Tomova et al., 2015). According to Grimaldi, increases in butyrate production, potentially positively affecting ASD, were detected in children with ASD following exclusion diets (Nankova et al., 2014; Grimaldi et al., 2018). Additionally, lower levels of amino acids (isoleucine, leucine, valine, alanine, and glutamine) and lactate were detected in the B-GOS group. The presence of amino acids in feces is associated with problems in gut barrier function (Marchesi et al., 2007).

##### 4.1.3.3 Gut microbiome modulation and GABA metabolism

A study of psychobiotics in a rodent ASD model, which was induced by oral propionic acid ingestion, proposed that *Lactobacillus bulgaricus* and *Bifidobacterium infantis* could ameliorate glutamate excitotoxicity, a major autistic feature in this model. The therapeutic effect of these psychobiotics might be due to the reduction of oxidative stress, restoration of the depleted GABA signaling pathway, and upregulation of the GABA receptor’s gene expression (Bin-Khattaf et al., 2022). Ingestion of *Lactobacillus rhamnosus* could connect bidirectional communication of the gut-brain axis, and it could regulate emotional behaviors by controlling the GABA receptor expression in the VN (Bravo et al., 2011). In 2018, the specific bacterial species of ASD were identified in *Shank3* knock-out mice, and this study suggested that oral *Lactobacillus reuteri* ingestion could decrease repetitive behaviors by up-regulation of the γ-aminobutyric acid (GABA)-related metabolism (Tabouy et al., 2018).

### 4.2 Therapeutic mechanisms/effects on ADHD

The etiology of ADHD is multifactorial. However, emerging research has shown the involvement of modulation in the gut microbiome and its promising effect on the clinical course of ADHD (Kalenik et al., 2021). In general, the effect of probiotic supplementation could act in both direct and indirect ways on ADHD, and ADHD-suffering children have higher GI severity index grade than healthy children (Ming et al., 2018). These GI symptoms can be relieved by adjustment of the gut microbial community *via* probiotic administration, however, Rianda’s randomized trial demonstrated that only one out of seven studies showed a positive effect of probiotics on cognitive function (Rianda et al., 2019).

#### 4.2.1 Neurotransmitters and metabolites

##### 4.2.1.1 Microbiome producing neurotransmitters

Bacteria can synthesize and respond to hormones and neurotransmitters. *Lactobacillus* species produce acetylcholine and GABA, *Bifidobacterium* species produce GABA, *Escherichia* produces norepinephrine, serotonin, and dopamine, *Streptococcus* and *Enterococcus* produce serotonin, and *Bacillus* species produce norepinephrine and dopamine (Galland, 2014). Other bacterial strains (*Lactococcus lactis* subsp. *cremoris*, *L. lactis* subsp. *lactis*, *Lactobacillus plantarum*, *Streptococcus thermophilus*, *Escherichia coli*, *Morganella morganii*, *Klebsiella pneumoniae*, and *Hafnia alvei*) produce serotonin (O’Mahony et al., 2015).

##### 4.2.1.2 Positive effects on dopaminergic and noradrenergic system

Various studies have suggested the dopamine hypothesis, which indicates that the enteric neurotransmitter release by nutrient intake could affect brain health and responses. And *Bacillus*, one of the representative bacteria to modulate the dopaminergic system, is known for its ability to produce dopamine and noradrenaline directly in the GI tract (Satti et al., 2023). Dysregulated dopaminergic and noradrenergic neurotransmission has been widely implicated in the pathophysiology of ADHD, and dopamine and norepinephrine play essential roles in behavioral, cognitive, and affective functions (Del Campo et al., 2011). In a study using patients with ADHD, *Bifidobacterium* was increased in patients with ADHD, which was linked with the enzyme involved in the dopamine precursor (phenylalanine) synthesis (Aarts et al., 2017)

##### 4.2.1.3 Synthesis of serotonin beyond BBB

Serotonin also plays a role in ADHD pathogenesis, however, it affects brain function not directly, but *via* the nervous system (Banerjee and Nandagopal, 2015; Hou et al., 2018). On the other hand, gut microbiota directly act a biological role on the brain by modulation tryptophan’s peripheral availability because tryptophan can cross the BBB and affect serotonin synthesis in CNS (Richard et al., 2009; Schwarcz and Stone, 2017).

##### 4.2.1.4 GABAergic system and GABA production

A recent experimental study has demonstrated that *Lactobacillus rhamnosus* regulates, *via* the VN, emotional behavior and the central GABAergic system, which is also associated with neuropsychiatric disorders (Enticott et al., 2010). According to Pärtty, the early supplementation of *Lactobacillus rhamnosus* GG decreases the risk of developing ADHD, and Liang-Jen Wang suggested that oral probiotic *Bifidobacterium bifidum* (Bf-688) improves the clinical symptoms of ADHD. In addition, food supplement treatments containing *Lactobacillus acidophilus* and *Bifidobacterium* improve the self-control and attention of children with ADHD (Pärtty et al., 2015; Wang et al., 2022). These results are thought to be due to the role of *Lactobacillus* and *Bifidobacterium* as producers of GABA, which is known to decrease in patients with ADHD (Yunes et al., 2016).

#### 4.2.2 Intermediate substances and metabolites

##### 4.2.2.1 Vagus nerve

The VN is the longest cranial nerve in the body, and it delivers electronic signals from the body (lungs, liver, heart, GI tract) to the brain through sensory fibers. And this connection administers the GI tract’s function *via* the metabolites from intestinal microorganisms. The VN is involved in functions such as mood control, immune response, and GI tract function *via* intestinal permeability and enteric reflex and influences the hypothalamic-pituitary-adrenal axis. Vagal afferent fibers sense microbiota signals indirectly through the diffusion of bacterial compounds, metabolites, or other cells located in the epithelium that relay luminal signals (Del Toro-Barbosa et al., 2020). The gut microbiome has the capacity to modulate the host’s emotional and behavioral responses by acting on vagal afferents.

##### 4.2.2.2 Short-chain fatty acids

Various host physiological metabolism could be regulated by SCFAs, in particular, gut barrier integrity, immune defense system, and lipid metabolism could be the major target of the SCFA. (Dalile et al., 2019). Moreover, SCFAs might directly influence neural function by reinforcing BBB integrity, modulating neurotransmission, influencing the levels of neurotrophic factors, and promoting memory consolidation. Increased evidence suggests a potential key role for SCFAs in gut-brain axis signaling (Silva et al., 2020). The ADHD group showed significantly lower concentrations of fecal acetate and butyrate than the control group, and various bacterial strains (*Bifidobacteria, L. salivarius, L. agilis, L. acidophilus, LGG, B. longum, B bifdum*, and *L. gasseri*) are known to increase SCFAs production (LeBlanc et al., 2017; Jung et al., 2022).

#### 4.2.3 Immune pathway and anti-inflammation

##### 4.2.3.1 Anti-inflammatory effects of probiotics

*Lactobacillus rhamnosus* GG is known to strengthen the gut permeability barrier by fortifying intestinal tight junctions, mucin layer thickness, and antigen-specific immunoglobulin A production (Asano et al., 2012). In particular, *L. rhamnosus GG* administrated participants showed a significant decrease in the serum levels of the pro-inflammatory cytokines (IL-6, IL-12 p70, and TNF-α). (Kumperscak et al., 2020).

## 5 FMT: rebuilding gut microecology

The definition of FMT is the transfer technique of a healthy donor’s fecal specimen to the GI tract of a recipient patient to reestablish the normal gut microbiome. This technique has been focused in recent years because of the technical advances in metagenomics sequencing and the growing understanding of its function. FMT has been demonstrated to be able to reconstruct a normally functioning microbial community, making it an accepted therapy with biological plausibility. Considering the effect of FMT on the reorganization of gut microbiota, it is considered to have the potential for the treatment of neurodevelopmental diseases such as ASD through the interaction of the MGB axis. It is necessary to determine the optimal composition of the microbiome to be used for FMT by clarifying the structure or functional profile of the microbes associated with improved clinical outcomes (Zhuang et al., 2019). Bacterial diversity and health-associated functions, such as colonization resistance, can be restored using FMT. In addition to bioactive compounds, FMT is also a source of microbes, such as phages. These components come together in a symbiotic community, allowing better colonization of the GI tract (Goldenberg et al., 2018). In terms of the gut microbiota, FMT is considered an untargeted intervention.

### 5.1 Therapeutic mechanisms and their effects on ASD

#### 5.1.1 Altering gut ecosystem

##### 5.1.1.1 Bacterial diversity

FMT could serve as a protective treatment for reconstructing the gut microbiota at both the phylum and genus levels and has a therapeutic effect on ASD symptoms and gastrointestinal disorders (Li et al., 2021). A modified FMT protocol for children with ASD, termed microbiota transfer therapy, appears to be a promising approach to alter the gut microbiome and improve GI and behavioral symptoms of ASD (Kang et al., 2017; Kang et al., 2019). This protocol improved GI and ASD symptoms, and the microbiome persisted for two years after treatment, suggesting a long-term impact. Important changes in the gut microbiota at the end of treatment were observed during follow-ups, including significant increases in bacterial diversity and relative abundance of *Bifidobacteria* and *Prevotella*.

##### 5.1.1.2 Engraftment of the donor microbiome

Li et al. showed that the gut microbial population of ASD children was altered by FMT with donor microbiota toward that of the healthy group. Especially, the FMT response significantly reduces the abundance of *Eubacterium coprostanoligenes* (Li et al., 2021). These data also indicated that decrement in the population of *Eubacterium coprostanoligenes* by FMT might be a curative technique for ASD symptoms and behaviors.

#### 5.1.2 Modulating neurotransmitters

##### 5.1.2.1 FMT alters the serum levels of neurotransmitters

Unlike probiotics, FMT refers to the transfer of the full spectrum of gut microbial communities containing more than 1,000 bacterial strains, and it might be more effective than psychobiotics in aspects of physiological regulation in the nervous system, endocrine system, and host behavior (Chen et al., 2022). In recent studies, FMT could exhibit a recovery effect on the serum levels of serotonin, GABA, and DA in the ASD cohort, which means that FMT might be an effective technique in regulating neurotransmitters *via* the MGB axis (Li et al., 2021). Moreover, FMT in the ASD cohort could decrease GABA and serotonin in serum, but the dopamine level was increased by FMT. It could be assumed that FMT may be an efficient approach to modulate neurotransmitter secretion for regulation of the central nerve *via* the MGB axis.

#### 5.1.3 Regulating immune responses

##### 5.1.3.1 Chemokines and microbiome

Alterations in the gut microbiota composition after FMT could significantly improve behavioral impairments and regulate immune responses in ASD. Chen and colleagues demonstrated that treatment using FMT with *in vitro* cultured healthy donor’s intestinal microbiota had a positive effect on ASD symptoms in mouse ASD model (Chen et al., 2020). They observed amelioration of anxiety actions and repetitive performance with lower serum levels of metabolites, such as GRO-α and MIP-1α, and a conversely higher level of MCP-3, RANTES, and Eotaxin. Additionally, family or genus levels of S24-7, *Clostridiaceae*, *Prevotella*, and *Candidatus Arthromitus* were key microbial taxa in FMT treatment, and serum levels of chemokines were related to the relative abundance of these taxa.

##### 5.1.3.2 Original donor vs *in vitro* cultured

In this study, both original donor microbiota transplantation and cultured microbiota transplantation improved behavioral abnormalities and chemokine disorders in an ASD mouse model and were effective in the modification of several key differential taxa in the gut microbial composition (Chen et al., 2020). These results of cultured microbiota transplantation suggest the possibility of using “donor-free FMT” and regulating the donor gut microbiota structure before transplantation during *in vitro* culture. The batch methods are fast, easy, and repeatable culturing techniques.

### 5.2 Therapeutic mechanisms/effects on ADHD

#### 5.2.1 Neuroprotective effects of the transplanted microbiome

A case report provides preliminary evidence regarding the use of FMT in a patient with *C. difficile* infection and ADHD. The authors suggested that gut microbiome modulation, particularly the gain or loss of specific microbial species and pathways involving the metabolism of SCFAs, tryptophan, and GABA, may merit further exploration as a potential therapeutic strategy for ADHD (Hooi et al., 2022). Among bacteria engrafted through FMT, *F. prausnitzii* may reduce neuroinflammation and alleviate ADHD symptoms. *F. prausnitzii* exhibits anti-inflammatory effects by increasing anti-inflammatory cytokines and decreasing inflammatory cytokines that promote neuroinflammation and development of ADHD. *L. ruminis* possesses genes contributing to the pentose phosphate pathway, which contributes to SCFA production. Engraftment of Lactobacillus genus may exert neuroprotection by producing anti-inflammatory SCFAs. (Basen and Kurrer, 2021).

## 6 Future perspectives and concluding remarks

Although the application of pre- and probiotics as psychobiotics remains promising, it is feasible that the effect of psychobiotics will be decreased over time due to the significant influence of environmental reasons occurred in child development (Grimaldi et al., 2018). Further studies should be needed to address the drug administration timing, the effect of different strain combinations, safety, and efficacy of probiotics. Furthermore, the novel therapeutic functions of the psychobiotics and commensal bacteria will be investigated in synthetic biology fields (Bin-Khattaf et al., 2022). For example, Korpela proposed that oral-fecal transplantation with diluted fecal samples from the maternal gut microbiome could restore normal gut microbiota in Cesarean-born infants (Korpela et al., 2020). Furthermore, a study demonstrated that using *E. coli* native to the target murine host to knock-in specific functions and apply them back to the host enabled the perpetual engraftment of transgenic bacteria in the intestine, which was demonstrated until the transformation stage of human borne *E. coli* (Asano et al., 2012). As such, various studies have focused on fortifying the modulating effects on the gut microbiome *via* adjustment at the molecular level.

The use of probiotics is feasible in children, and short-term supplementation has been shown to be safe. However, the long-term effects of repeated applications on the gut microbiome and the safety concerns of treatment are unknown. The actual efficacy of FMT has been proved by various studies using diverse animal models. However, Safety is the most important aspect in the FMT study because most ASD patients are children. In previous studies, the oral ingestion of human fecal suspensions was considered an unpleasant experience for patients and might cause side effects, including extra ingestion with acid inhibitors. As it has been known that a colon-release capsule coated with acid-resistant hydroxypropyl cellulose is the best formulation for patients (Kang et al., 2019).

This review summarizes the current knowledge on the positive effects and potential pathways of promising therapeutic interventions, including psychobiotic supplementation and modulation of the gut microbiome to improve the GI and behavioral symptoms of patients with ASD or ADHD. Development of the gut microbiome in early life plays an important role in the overall well-being of humans. Numerous studies have demonstrated that early alterations in the gut microbiome are closely related to neurodevelopmental disorders, such as ASD and ADHD. Nevertheless, the ambiguous and equivocal evidence of clinical studies makes it difficult to believe the therapeutic method targeting the MGB axis. To better understand the role of the gut microbiome in heterogeneous and complex ASD/ADHD pathogenesis, double-blind, randomized, controlled trials and treatments tailored to individual characteristics and the host microbiome are recommended. In particular, the process of intestinal microbiota colonization and establishment in the early stage of life is crucially affected by maternal conditions/diseases, mode of delivery, and exposure to antibiotics. Therefore, future studies are needed to determine more accurate therapeutic targets in immune, metabolic, endocrine, and neural pathways by mechanism validation through culturomics experiments of mainly modulated microbial populations and metabolomic analysis of the mother’s skin, vagina, gut microbiota, and infant gut environments.

## Author contributions

All authors researched the data for this article, made substantial contributions to discussions of the content, wrote the article, and reviewed and/or edited the manuscript prior to submission.

## References

[B1] Aarts E. Ederveen T. H. Naaijen J. Zwiers M. P. Boekhorst J. Timmerman H. M.. (2017). Gut microbiome in ADHD and its relation to neural reward anticipation. PloS One 12 (9), e0183509. doi: 10.1371/journal.pone.0183509 28863139 PMC5581161

[B2] Adıgüzel E. Çiçek B. Ünal G. Aydın M. F. Barlak-Keti D. (2022). Probiotics and prebiotics alleviate behavioral deficits, inflammatory response, and gut dysbiosis in prenatal VPA-induced rodent model of autism. Physiol. Behav. 256, 113961. doi: 10.1016/j.physbeh.2022.113961 36100109

[B3] Alonazi M. Ben Bacha A. Al Suhaibani A. Almnaizel A. T. Aloudah H. S. El-Ansary A. (2022). Psychobiotics improve propionic acid-induced neuroinflammation in juvenile rats, rodent model of autism. Transl. Neurosci. 13 (1), 292–300. doi: 10.1515/tnsci-2022-0226 36133749 PMC9462542

[B4] Aoki-Yoshida A. Saito S. Tsuruta T. Ohsumi A. Tsunoda H. Sonoyama K. (2017). Exosomes isolated from sera of mice fed lactobacillus strains affect inflammatory cytokine production in macrophages inávitro. Biochem. Biophys. Res. Commun. 489 (2), 248–254. doi: 10.1016/j.bbrc.2017.05.152 28559134

[B5] Arnold L. E. Luna R. A. Williams K. Chan J. Parker R. A. Wu Q.. (2019). Probiotics for gastrointestinal symptoms and quality of life in autism: a placebo-controlled pilot trial. J. Child Adolesc. Psychopharmacol. 29 (9), 659–669. doi: 10.1089/cap.2018.0156 31478755 PMC7364307

[B6] Arrieta M. C. Finlay B. B. (2012). The commensal microbiota drives immune homeostasis. Front. Immunol. 3. doi: 10.3389/fimmu.2012.00033 PMC3341987 22566917

[B7] Arrieta M. C. Stiemsma L. T. Amenyogbe N. Brown E. M. Finlay B. (2014). The intestinal microbiome in early life: health and disease. Front. Immunol. 5. doi: 10.3389/fimmu.2014.00427 PMC4155789 25250028

[B8] Asano Y. Hiramoto T. Nishino R. Aiba Y. Kimura T. Yoshihara K.. (2012). Critical role of gut microbiota in the production of biologically active, free catecholamines in the gut lumen of mice. Am. J. Physiol. Gastrointest. Liver Physiol. 303 (11), G1288–G1295. doi: 10.1152/ajpgi.00341.2012 23064760

[B9] Bäckhed F. Roswall J. Peng Y. Feng Q. Jia H. Kovatcheva-Datchary P.. (2015). Dynamics and stabilization of the human gut microbiome during the first year of life. Cell Host Microbe 17 (5), 690–703. doi: 10.1016/j.chom.2015.04.004 25974306

[B10] Banerjee E. Nandagopal K. (2015). Does serotonin deficit mediate susceptibility to ADHD? Neurochem. Int. 82, 52–68. doi: 10.1016/j.neuint.2015.02.001 25684070

[B11] Basen M. Kurrer S. E. (2021). A close look at pentose metabolism of gut bacteria. FEBS J. 288 (6), 1804–1808. doi: 10.1111/febs.15575 33063458

[B12] Bermúdez-Humarán L. G. Salinas E. Ortiz G. G. Ramirez-Jirano L. J. Morales J. A. Bitzer-Quintero O. K. (2019). From probiotics to psychobiotics: live beneficial bacteria which act on the brain-gut axis. Nutrients 11 (4), 890. doi: 10.3390/nu11040890 31010014 PMC6521058

[B13] Bin-Khattaf R. M. Alonazi M. A. Al-Dbass A. M. Almnaizel A. T. Aloudah H. S. Soliman D. A.. (2022). Probiotic ameliorating effects of altered GABA/glutamate signaling in a rodent model of autism. Metabolites 12 (8), 720. doi: 10.3390/metabo12080720 36005593 PMC9416367

[B14] Bravo J. A. Forsythe P. Chew M. V. Escaravage E. Savignac H. M. Dinan T. G.. (2011). Ingestion of lactobacillus strain regulates emotional behavior and central GABA receptor expression in a mouse *via* the vagus nerve. Proc. Nat. Acad. Sci. 108 (38), 16050–16055. doi: 10.1073/pnas.110299910 21876150 PMC3179073

[B15] Brikell I. Burton C. Mota N. R. Martin J. (2021). Insights into attention-deficit/hyperactivity disorder from recent genetic studies. Psychol. Med. 51 (13), 2274–2286. doi: 10.1017/S0033291721000982 33814023

[B16] Bundgaard-Nielsen C. Knudsen J. Leutscher P. D. Lauritsen M. B. Nyegaard M. Hagstrøm S.. (2020). Gut microbiota profiles of autism spectrum disorder and attention deficit/hyperactivity disorder: a systematic literature review. Gut Microbes 11 (5), 1172–1187. doi: 10.1080/19490976.2020.1748258 32329656 PMC7524304

[B17] Bundgaard-Nielsen C. Lauritsen M. B. Knudsen J. K. Rold L. S. Larsen M. H. Hindersson P.. (2023). Children and adolescents with attention deficit hyperactivity disorder and autism spectrum disorder share distinct microbiota compositions. Gut Microbes 15 (1), 2211923. doi: 10.1080/19490976.2023.2211923 37199526 PMC10197996

[B18] Cai Z. He W. Zhuang F. J. Chen Y. (2019). The role of high high-sensitivity c-reactive protein levels at admission on poor prognosis after acute ischemic stroke. Int. J. Neurosci. 129 (5), 423–429. doi: 10.1080/00207454.2018.1538139 30332913

[B19] Carlson A. L. Xia K. Azcarate-Peril M. A. Goldman B. D. Ahn M. Styner M. A.. (2018). Infant gut microbiome associated with cognitive development. Biol. Psychiatry 83 (2), 148–159. doi: 10.1016/j.biopsych.2017.06.021 28793975 PMC5724966

[B20] Chen K. Fu Y. Wang Y. Liao L. Xu H. Zhang A.. (2020). Therapeutic effects of the *in vitro* cultured human gut microbiota as transplants on altering gut microbiota and improving symptoms associated with autism spectrum disorder. Microb. Ecol. 80, 475–486. doi: 10.1007/s00248-020-01494-w 32100127

[B21] Chen Y. Xueying Z. Jiaqu C. Qiyi C. Huanlong Q. Ning L.. (2022). FTACMT study protocol: a multicentre, double-blind, randomised, placebo-controlled trial of faecal microbiota transplantation for autism spectrum disorder. BMJ Open 12 (1), e051613. doi: 10.1136/bmjopen-2021-051613 PMC8804636 35105621

[B22] Cheng L. H. Liu Y. W. Wu C. C. Wang S. Tsai Y. C. (2019). Psychobiotics in mental health, neurodegenerative and neurodevelopmental disorders. J. Food Drug Anal. 27 (3), 632–648. doi: 10.1016/j.jfda.2019.01.002 31324280 PMC9307042

[B23] Cheroni C. Caporale N. Testa G. (2020). Autism spectrum disorder at the crossroad between genes and environment: contributions, convergences, and interactions in ASD developmental pathophysiology. Mol. Autism. 11 (1), 69. doi: 10.1186/s13229-020-00370-1 32912338 PMC7488083

[B24] Choi G. B. Yim Y. S. Wong H. Kim S. Kim H. Kim S. V.. (2016). The maternal interleukin-17a pathway in mice promotes autism-like phenotypes in offspring. Science 351 (6276), 933–939. doi: 10.1126/science.aad0314 26822608 PMC4782964

[B25] Cicenia A. Santangelo F. Gambardella L. Pallotta L. Iebba V. Scirocco A.. (2016). Protective role of postbiotic mediators secreted by lactobacillus rhamnosus GG versus lipopolysaccharide-induced damage in human colonic smooth muscle cells. J. Clin. Gastroenterol. 50, S140–S144. doi: 10.1097/MCG.0000000000000681 27741159

[B26] Clarke T. C. (2018). The use of complementary health approaches among US adults with a recent cancer diagnosis. J. Altern. Complement. Med. 24 (2), 139–145. doi: 10.1089/acm.2016.0182 28930475 PMC5820530

[B27] Cryan J. F. O'Riordan K. J. Cowan C. S. Sandhu K. V. Bastiaanssen T. F. Boehme M.. (2019). The microbiota-gut-brain axis. Physiol. Rev. 99 (4), 1877–2013. doi: 10.1152/physrev.00018.2018 31460832

[B28] Dalile B. Van Oudenhove L. Vervliet B. Verbeke K. (2019). The role of short-chain fatty acids in microbiota–gut–brain communication. Nat. Rev. Gastroenterol. Hepatol. 16 (8), 461–478. doi: 10.1038/s41575-019-0157-3 31123355

[B29] Daliri E. B. M. Oh D. H. Lee B. H. (2016). Psychobiotics; a promise for neurodevelopmental therapy. J. Probiotics Health 4, 1e4. doi: 10.4172/2329-8901.1000146

[B30] De Angelis M. Piccolo M. Vannini L. Siragusa S. De Giacomo A. Serrazzanetti D. I.. (2013). Fecal microbiota and metabolome of children with autism and pervasive developmental disorder not otherwise specified. PloS One 8 (10), e76993. doi: 10.1371/journal.pone.0076993 24130822 PMC3793965

[B31] Del Campo N. Chamberlain S. R. Sahakian B. J. Robbins T. W. (2011). The roles of dopamine and noradrenaline in the pathophysiology and treatment of attention-deficit/hyperactivity disorder. Biol. Psychiatry 69 (12), e145–e157. doi: 10.1016/j.biopsych.2011.02.036 21550021

[B32] Del Toro-Barbosa M. Hurtado-Romero A. Garcia-Amezquita L. E. García-Cayuela T. (2020). Psychobiotics: mechanisms of action, evaluation methods and effectiveness in applications with food products. Nutrients 12 (12), 3896. doi: 10.3390/nu12123896 33352789 PMC7767237

[B33] Dinan T. G. Borre Y. E. Cryan J. F. (2014). Genomics of schizophrenia: time to consider the gut microbiome? Mol. Psychiatry 19 (12), 1252–1257. doi: 10.1038/mp.2014.93 25288135

[B34] Dinan T. G. Cryan J. F. (2017). Brain–gut–microbiota axis–mood, metabolism and behaviour. Nat. Rev. Gastroenterol. Hepatol. 14 (2), 69–70. doi: 10.1038/nrgastro.2016.200 28053341

[B35] Enticott P. G. Rinehart N. J. Tonge B. J. Bradshaw J. L. Fitzgerald P. B. (2010). A preliminary transcranial magnetic stimulation study of cortical inhibition and excitability in high-functioning autism and asperger disorder. Dev. Med. Child Neurol. 52 (8), e179–e183. doi: 10.1111/j.1469-8749.2010.03665.x 20370810

[B36] Erkosar B. Storelli G. Defaye A. Leulier F. (2013). Host-intestinal microbiota mutualism:”learning on the fly”. Cell Host Microbe 13 (1), 8–14. doi: 10.1016/j.chom.2012.12.004 23332152

[B37] Faraone S. V. Larsson H. (2019). Genetics of attention deficit hyperactivity disorder. Mol. Psychiatry 24 (4), 562–575. doi: 10.1038/s41380-018-0070-0 29892054 PMC6477889

[B38] Fong F. L. Y. Kirjavainen P. V. El-Nezami H. (2016). Immunomodulation of lactobacillus rhamnosus GG (LGG)-derived soluble factors on antigen-presenting cells of healthy blood donors. Sci. Rep. 6 (1), 22845. doi: 10.1038/srep22845 26961406 PMC4785377

[B39] Fowlie G. Cohen N. Ming X. (2018). The perturbance of microbiome and gut-brain axis in autism spectrum disorders. Int. J. Mol. Sci. 19 (8), 2251. doi: 10.3390/ijms19082251 30071612 PMC6121241

[B40] Galland L. (2014). The gut microbiome and the brain. J. Med. Food 17 (12), 1261–1272. doi: 10.1089/jmf.2014.7000 25402818 PMC4259177

[B41] Ghanaatgar M. Taherzadeh S. Ariyanfar S. Jahromi S. R. Martami F. Gharaei J. M.. (2022). Probiotic supplement as an adjunctive therapy with Ritalin for treatment of attention-deficit hyperactivity disorder symptoms in children: a double-blind placebo-controlled randomized clinical trial. Nutr. Food Sci. 53 (1), 19–34. doi: 10.1108/NFS-12-2021-0388

[B42] Goldenberg S. D. Batra R. Beales I. Digby-Bell J. L. Irving P. M. Kellingray L.. (2018). Comparison of different strategies for providing fecal microbiota transplantation to treat patients with recurrent clostridium difficile infection in two English hospitals: a review. Infect. Dis. Ther. 7, 71–86. doi: 10.1007/s40121-018-0189-y 29450831 PMC5840108

[B43] Grimaldi R. Gibson G. R. Vulevic J. Giallourou N. Castro-Mejía J. L. Hansen L. H.. (2018). A prebiotic intervention study in children with autism spectrum disorders (ASDs). Microbiome 6 (1), 1–13. doi: 10.1186/s40168-018-0523-3 30071894 PMC6091020

[B44] Grossi E. Melli S. Dunca D. Terruzzi V. (2016). Unexpected improvement in core autism spectrum disorder symptoms after long-term treatment with probiotics. SAGE open med. Case Rep. 4, 2050313X16666231. doi: 10.1177/2050313X16666231 PMC5006292 27621806

[B45] Guidetti C. Salvini E. Viri M. Deidda F. Amoruso A. Visciglia A.. (2022). Randomized double-blind crossover study for evaluating a probiotic mixture on gastrointestinal and behavioral symptoms of autistic children. J.Clin. Med. 11 (18), 5263. doi: 10.3390/jcm11185263 36142909 PMC9504504

[B46] Hooi S. L. Dwiyanto J. Rasiti H. Toh K. Y. Wong R. K. M. Lee J. W. J. (2022). A case report of improvement on ADHD symptoms after fecal microbiota transplantation with gut microbiome profiling pre-and post-procedure. Curr. Med. Res. Opin. 38 (11), 1977–1982. doi: 10.1080/03007995.2022.2129232 36164761

[B47] Hou Y. W. Xiong P. Gu X. Huang X. Wang M. Wu J. (2018). Association of serotonin receptors with attention deficit hyperactivity disorder: a systematic review and meta-analysis. Curr. Med. Sci. 38, 538–551. doi: 10.1007/s11596-018-1912-3 30074224

[B48] Hsiao E. Y. McBride S. W. Hsien S. Sharon G. Hyde E. R. McCue T.. (2013). Microbiota modulate behavioral and physiological abnormalities associated with neurodevelopmental disorders. Cell. 155 (7), 1451–1463. doi: 10.1016/j.cell.2013.11.024 24315484 PMC3897394

[B49] Jena A. Montoya C. A. Mullaney J. A. Dilger R. N. Young W. McNabb W. C.. (2020). Gut-brain axis in the early postnatal years of life: a developmental perspective. Front. Iinteg. Neurosci. 14. doi: 10.3389/fnint.2020.00044 PMC7419604 32848651

[B50] Jung T. H. Hwang H. J. Han K. S. (2022). Correlation of attention deficit hyperactivity disorder with gut microbiota according to the dietary intake of Korean elementary school students. PloS One 17 (9), e0275520. doi: 10.1371/journal.pone.0275520 36178961 PMC9524712

[B51] Kalenik A. Kardaś K. Rahnama A. Sirojć K. Wolańczyk T. (2021). Gut microbiota and probiotic therapy in ADHD: a review of current knowledge. Prog. Neuropsychopharmacol. Biol. Psychiatry 110, 110277. doi: 10.1016/j.pnpbp.2021.110277 33561522

[B52] Kałużna-Czaplińska J. Błaszczyk S. (2012). The level of arabinitol in autistic children after probiotic therapy. Nutrition. 28 (2), 124–126. doi: 10.1016/j.nut.2011.08.002 22079796

[B53] Kang D. W. Adams J. B. Coleman D. M. Pollard E. L. Maldonado J. McDonough-Means S.. (2019). Long-term benefit of microbiota transfer therapy on autism symptoms and gut microbiota. Sci. Rep. 9 (1), 5821. doi: 10.1038/s41598-019-42183-0 30967657 PMC6456593

[B54] Kang D. W. Adams J. B. Gregory A. C. Borody T. Chittick L. Fasano A.. (2017). Microbiota transfer therapy alters gut ecosystem and improves gastrointestinal and autism symptoms: an open-label study. Microbiome 5 (1), 1–16. doi: 10.1186/s40168-016-0225-7 28122648 PMC5264285

[B55] Kho Z. Y. Lal S. K. (2018). The human gut microbiome–a potential controller of wellness and disease. Front. Microbiol. 9, 1835. doi: 10.3389/fmicb.2018.01835 30154767 PMC6102370

[B56] Kong X. J. Liu J. Liu K. Koh M. Sherman H. Liu S.. (2021). Probiotic and oxytocin combination therapy in patients with autism spectrum disorder: a randomized, double-blinded, placebo-controlled pilot trial. Nutrients. 13 (5), 1552. doi: 10.3390/nu13051552 34062986 PMC8147925

[B57] Korpela K. Helve O. Kolho K. L. Saisto T. Skogberg K. Dikareva E.. (2020). Maternal fecal microbiota transplantation in cesarean-born infants rapidly restores normal gut microbial development: a proof-of-concept study. Cell 183 (2), 324–334. doi: 10.1016/j.cell.2020.08.047 33007265

[B58] Kumperscak H. G. Gricar A. Ülen I. Micetic-Turk D. (2020). A pilot randomized control trial with the probiotic strain lactobacillus rhamnosus GG (LGG) in ADHD: children and adolescents report better health-related quality of life. Front. Psychiatry 11. doi: 10.3389/fpsyt.2020.00181 PMC7092625 32256407

[B59] Kwak M. J. Tan P. L. Oh J. K. Chae K. S. Kim J. Kim S. H. (2021). The effects of multispecies probiotic formulations on growth performance, hepatic metabolism, intestinal integrity and fecal microbiota in growing-finishing pigs. Animal Feed Science and Technology 274, 114833.

[B60] LeBlanc J. G. Chain F. Martín R. Bermúdez-Humarán L. G. Courau S. Langella P. (2017). Beneficial effects on host energy metabolism of short-chain fatty acids and vitamins produced by commensal and probiotic bacteria. Microb. Cell Factories 16 (1), 1–10. doi: 10.1186/s12934-017-0691-z PMC5423028 28482838

[B61] Li N. Chen H. Cheng Y. Xu F. Ruan G. Ying S.. (2021). Fecal microbiota transplantation relieves gastrointestinal and autism symptoms by improving the gut microbiota in an open-label study. Front. Cell. Infect. Microbiol. 11. doi: 10.3389/fcimb.2021.759435 PMC8560686 34737978

[B62] Liu Y. W. Liong M. T. Chung Y. C. E. Huang H. Y. Peng W. S. Cheng Y. F.. (2019). Effects of lactobacillus plantarum PS128 on children with autism spectrum disorder in Taiwan: a randomized, double-blind, placebo-controlled trial. Nutrients. 11 (4), 820. doi: 10.3390/nu11040820 30979038 PMC6521002

[B63] Liu S. Rao S. Xu Y. Li J. Huang H. Zhang X.. (2020). Identifying common genome-wide risk genes for major psychiatric traits. Hum. Genet. 139, 185–198. doi: 10.1007/s00439-019-02096-4 31813014

[B64] Marchesi J. R. Holmes E. Khan F. Kochhar S. Scanlan P. Shanahan F.. (2007). Rapid and noninvasive metabonomic characterization of inflammatory bowel disease. J. Proteome Res. 6 (2), 546–551. doi: 10.1021/pr060470d 17269711

[B65] Mensi M. M. Rogantini C. Marchesi M. Borgatti R. Chiappedi M. (2021). *Lactobacillus plantarum* PS128 and other probiotics in children and adolescents with autism spectrum disorder: a real-world experience. Nutrients. 13 (6), 2036. doi: 10.3390/nu13062036 34198499 PMC8231766

[B66] Ming X. Chen N. Ray C. Brewer G. Kornitzer J. Steer R. A. (2018). A gut feeling: a hypothesis of the role of the microbiome in attention-deficit/hyperactivity disorders. Child Neurol. Open 5, 2329048X18786799. doi: 10.1177/2329048X18786799 PMC6047248 30023407

[B67] Mintál K. Tóth A. Hormay E. Kovács A. László K. Bufa A.. (2022). Novel probiotic treatment of autism spectrum disorder associated social behavioral symptoms in two rodent models. Sci. Rep. 12 (1), 5399. doi: 10.1038/s41598-022-09350-2 35354898 PMC8967893

[B68] Miyazawa K. Yoda K. Kawase M. Harata G. He F. (2015). Influence of orally administered lactobacillus GG on respiratory immune response in a murine model of diet-induced obesity. Microbiol. Immunol. 59 (2), 99–103. doi: 10.1111/1348-0421.12226 25643737

[B69] Morais L. H. Schreiber H. L. IV Mazmanian S. K. (2021). The gut microbiota–brain axis in behaviour and brain disorders. Nat. Rev. Microbiol. 19 (4), 241–255. doi: 10.1038/s41579-020-00460-0 33093662

[B70] Nagpal J. Cryan J. F. (2021). Microbiota-brain interactions: moving toward mechanisms in model organisms. Neuron 109 (24), 3930–3953. doi: 10.1016/j.neuron.2021.09.036 34653349

[B71] Nankova B. B. Agarwal R. MacFabe D. F. La Gamma E. F. (2014). Enteric bacterial metabolites propionic and butyric acid modulate gene expression, including CREB-dependent catecholaminergic neurotransmission, in PC12 cells-possible relevance to autism spectrum disorders. PloS One 9 (8), e103740. doi: 10.1371/journal.pone.0103740 25170769 PMC4149359

[B72] Niemarkt H. J. De Meij T. G. van Ganzewinkel C. J. de Boer N. K. Andriessen P. Hütten M. C.. (2019). Necrotizing enterocolitis, gut microbiota, and brain development: role of the brain-gut axis. Neonatology 115 (4), 423–431. doi: 10.1159/000497420 30974443 PMC6604259

[B73] O’Mahony S. M. Clarke G. Borre Y. E. Dinan T. G. Cryan J. F. (2015). Serotonin, tryptophan metabolism and the brain-gut-microbiome axis. Behav. Brain Res. 277, 32–48. doi: 10.1016/j.bbr.2014.07.027 25078296

[B74] Pärtty A. Kalliomäki M. Wacklin P. Salminen S. Isolauri E. (2015). A possible link between early probiotic intervention and the risk of neuropsychiatric disorders later in childhood: a randomized trial. Pediatr. Res. 77 (6), 823–828. doi: 10.1038/pr.2015.51 25760553

[B75] Perez-Burgos A. Wang B. Mao Y. K. Mistry B. Neufeld K. A. M. Bienenstock J.. (2013). Psychoactive bacteria *Lactobacillus rhamnosus* (JB-1) elicits rapid frequency facilitation in vagal afferents. Am. J. Physiol. Gastrointest. Liver Physiol. 304 (2), G211–G220. doi: 10.1152/ajpgi.00128.2012 23139216

[B76] Polanczyk G. De Lima M. S. Horta B. L. Biederman J. Rohde L. A. (2007). The worldwide prevalence of ADHD: a systematic review and metaregression analysis. Am. J. Psychiatry 164 (6), 942–948. doi: 10.1176/appi.ajp.164.6.942 17541055

[B77] Prosperi M. Santocchi E. Guiducci L. Frinzi J. Morales M. A. Tancredi R.. (2022). Interventions on microbiota: where do we stand on a gut–brain link in autism? A Systematic Review. Nutrients. 14 (3), 462. doi: 10.3390/nu14030462 35276821 PMC8839651

[B78] Rianda D. Agustina R. Setiawan E. A. Manikam N. R. M. (2019). Effect of probiotic supplementation on cognitive function in children and adolescents: a systematic review of randomised trials. Benef. Microbes 10 (8), 873–882. doi: 10.3920/BM2019.0068 31965841

[B79] Richard D. M. Dawes M. A. Mathias C. W. Acheson A. Hill-Kapturczak N. Dougherty D. M. (2009). L-tryptophan: basic metabolic functions, behavioral research and therapeutic indications. Int. J. Tryptophan Res. 2, IJTR–S2129. doi: 10.4137/ijtr.s2129 PMC2908021 20651948

[B80] Rommelse N. N. Franke B. Geurts H. M. Hartman C. A. Buitelaar J. K. (2010). Shared heritability of attention-deficit/hyperactivity disorder and autism spectrum disorder. Eur. Child Adolesc. Psychiatry 19, 281–295. doi: 10.1007/s00787-010-0092-x 20148275 PMC2839489

[B81] Ronan V. Yeasin R. Claud E. C. (2021). Childhood development and the microbiome–the intestinal microbiota in maintenance of health and development of disease during childhood development. Gastroenterology. 160 (2), 495–506. doi: 10.1053/j.gastro.2020.08.065 33307032 PMC8714606

[B82] Sanctuary M. R. Kain J. N. Chen S. Y. Kalanetra K. Lemay D. G. Rose D. R.. (2019). Pilot study of probiotic/colostrum supplementation on gut function in children with autism and gastrointestinal symptoms. PloS One 14 (1), e0210064. doi: 10.1371/journal.pone.0210064 30625189 PMC6326569

[B83] Santocchi E. Guiducci L. Prosperi M. Calderoni S. Gaggini M. Apicella F.. (2020). Effects of probiotic supplementation on gastrointestinal, sensory and core symptoms in autism spectrum disorders: a randomized controlled trial. Front. Psychiatry 11. doi: 10.3389/fpsyt.2020.550593 PMC7546872 33101079

[B84] Sarkar A. Lehto S. M. Harty S. Dinan T. G. Cryan J. F. Burnet P. W. (2016). Psychobiotics and the manipulation of bacteria–gut–brain signals. Trends Neurosci. 39 (11), 763–781. doi: 10.1016/j.tins.2016.09.002 27793434 PMC5102282

[B85] Satti S. Palepu M. S. K. Singh A. A. Jaiswal Y. Dash S. P. Gajula S. N. R.. (2023). Anxiolytic-and antidepressant-like effects of bacillus coagulans unique IS-2 mediate *via* reshaping of microbiome gut-brain axis in rats. Neurochem. Int. 163, 105483. doi: 10.1016/j.neuint.2023.105483 36641109

[B86] Schwarcz R. Stone T. W. (2017). The kynurenine pathway and the brain: challenges, controversies and promises. Neuropharmacology 112, 237–247. doi: 10.1016/j.neuropharm.2016.08.003 27511838 PMC5803785

[B87] Selma-Royo M. Calatayud Arroyo M. García-Mantrana I. Parra-Llorca A. Escuriet R. Martínez-Costa C.. (2020). Perinatal environment shapes microbiota colonization and infant growth: impact on host response and intestinal function. Microbiome 8 (1), 1–19. doi: 10.1186/s40168-020-00940-8 33228771 PMC7685601

[B88] Senn V. Bassler D. Choudhury R. Scholkmann F. Righini-Grunder F. Vuille-dit-Bille R. N.. (2020). Microbial colonization from the fetus to early childhood–a comprehensive review. Front. Cell. Infect. Microbiol. 10. doi: 10.3389/fcimb.2020.573735 PMC7661755 33194813

[B89] Sepehrmanesh Z. Shahzeidi A. Mansournia M. A. Ghaderi A. Ahmadvand A. (2021). Clinical and metabolic reaction to probiotic supplement in children suffering attention-deficit hyperactivity disorder: a randomized, double-blind, placebo-controlled experiment. Int. Arch. Health Sci. 8 (2), 90. doi: 10.4103/iahs.iahs_112_20

[B90] Sgritta M. Dooling S. W. Buffington S. A. Momin E. N. Francis M. B. Britton R. A.. (2019). Mechanisms underlying microbial-mediated changes in social behavior in mouse models of autism spectrum disorder. Neuron 101 (2), 246–259. doi: 10.1016/j.neuron.2018.11.018 30522820 PMC6645363

[B91] Shaaban S. Y. El Gendy Y. G. Mehanna N. S. El-Senousy W. M. El-Feki H. S. Saad K.. (2018). The role of probiotics in children with autism spectrum disorder: a prospective, open-label study. Nutr. Neurosci. 21 (9), 676–681. doi: 10.1080/1028415X.2017.1347746 28686541

[B92] Sharma R. Gupta D. Mehrotra R. Mago P. (2021). Psychobiotics: the next-generation probiotics for the brain. Curr. Microbiol. 78, 449–463. doi: 10.1007/s00284-020-02289-5 33394083

[B93] Shaw W. Kassen E. Chaves E. (1995). Increased urinary excretion of analogs of Krebs cycle metabolites and arabinose in two brothers with autistic features. Clin. Chem. 41 (8), 1094–1104. doi: 10.1093/clinchem/41.8.1094 7628083

[B94] Silva Y. P. Bernardi A. Frozza R. L. (2020). The role of short-chain fatty acids from gut microbiota in gut-brain communication. Front. Endocrinol. 11. doi: 10.3389/fendo.2020.00025 PMC7005631 32082260

[B95] Skott E. Yang L. L. Stiernborg M. Söderström Å. Rüegg J. Schalling M.. (2020). Effects of a synbiotic on symptoms, and daily functioning in attention deficit hyperactivity disorder–a double-blind randomized controlled trial. Brain Behav. Immun. 89, 9–19. doi: 10.1016/j.bbi.2020.05.056 32497779

[B96] Stiemsma L. T. Michels K. B. (2018). The role of the microbiome in the developmental origins of health and disease. Pediatrics 141 (4), e20172437. doi: 10.1542/peds.2017-2437 29519955 PMC5869344

[B97] Sukmajaya A. C. Lusida M. I. Setiawati Y. (2021). Systematic review of gut microbiota and attention-deficit hyperactivity disorder (ADHD). Ann. Gen. Psychiatry 20 (1), 1–12. doi: 10.1186/s12991-021-00330-w 33593384 PMC7888126

[B98] Tabouy L. Getselter D. Ziv O. Karpuj M. Tabouy T. Lukic I.. (2018). Dysbiosis of microbiome and probiotic treatment in a genetic model of autism spectrum disorders. Brain Behav. Immun. 73, 310–319. doi: 10.1016/j.bbi.2018.05.015 29787855

[B99] Tick B. Bolton P. Happé F. Rutter M. Rijsdijk F. (2016). Heritability of autism spectrum disorders: a meta-analysis of twin studies. J. Child Psychol. Psychiatry 57 (5), 585–595. doi: 10.1111/jcpp.12499 26709141 PMC4996332

[B100] Tomova A. Husarova V. Lakatosova S. Bakos J. Vlkova B. Babinska K.. (2015). Gastrointestinal microbiota in children with autism in Slovakia. Physiol. Behav. 138, 179–187. doi: 10.1016/j.physbeh.2014.10.033 25446201

[B101] Vuong H. E. Hsiao E. Y. (2017). Emerging roles for the gut microbiome in autism spectrum disorder. Biol. Psychiatry 81 (5), 411–423. doi: 10.1016/j.biopsych.2016.08.024 27773355 PMC5285286

[B102] Wang H. Gao K. Wen K. Allen I. C. Li G. Zhang W.. (2016). Lactobacillus rhamnosus GG modulates innate signaling pathway and cytokine responses to rotavirus vaccine in intestinal mononuclear cells of gnotobiotic pigs transplanted with human gut microbiota. BMC Microbiol. 16 (1), 1–14. doi: 10.1186/s12866-016-0727-2 27301272 PMC4908676

[B103] Wang L. J. Yang C. Y. Kuo H. C. Chou W. J. Tsai C. S. Lee S. Y. (2022). Effect of bifidobacterium bifidum on clinical characteristics and gut microbiota in attention-Deficit/Hyperactivity disorder. J. Pers. Med. 12 (2), 227. doi: 10.3390/jpm12020227 35207715 PMC8877879

[B104] Yunes R. A. Poluektova E. U. Dyachkova M. S. Klimina K. M. Kovtun A. S. Averina O. V.. (2016). GABA production and structure of gadB/gadC genes in lactobacillus and bifidobacterium strains from human microbiota. Anaerobe 42, 197–204. doi: 10.1016/j.anaerobe.2016.10.011 27794467

[B105] Zheng D. Liwinski T. Elinav E. (2020). Interaction between microbiota and immunity in health and disease. Cell Res. 30 (6), 492–506. doi: 10.1038/s41422-020-0332-7 32433595 PMC7264227

[B106] Zheng Y. Verhoeff T. A. Perez Pardo P. Garssen J. Kraneveld A. D. (2020). The gut-brain axis in autism spectrum disorder: a focus on the metalloproteases ADAM10 and ADAM17. Int. J. Mol. Sci. 22 (1), 118. doi: 10.3390/ijms22010118 33374371 PMC7796333

[B107] Zhou A. Cao X. Mahaganapathy V. Azaro M. Gwin C. Wilson S.. (2023). Common genetic risk factors in ASD and ADHD co-occurring families. Hum. Genet. 142 (2), 217–230. doi: 10.1007/s00439-022-02496-z 36251081 PMC10177627

[B108] Zhuang L. Chen H. Zhang S. Zhuang J. Li Q. Feng Z. (2019). Intestinal microbiota in early life and its implications on childhood health. Genomics Proteomics Bioinf. 17 (1), 13–25. doi: 10.1016/j.gpb.2018.10.002 PMC6522475 30986482

